# Preparation, Characterization and Gelation of a Fungal Nano Chitin Derived from *Hericium erinaceus* Residue

**DOI:** 10.3390/polym14030474

**Published:** 2022-01-25

**Authors:** Jing Liao, Huihua Huang

**Affiliations:** 1 College of Food and Biological Engineering, Chengdu University, Chengdu 610106, China; liaojing1228@cdu.edu.cn; 2 School of Food Science and Engineering, South China University of Technology, Guangzhou 510641, China

**Keywords:** nano chitin, TEMPO-mediated oxidation, dispersion stability, hydrogels

## Abstract

Nano chitin is a promising biocompatible material with wide applications. In this work, a fungal-derived nano chitin was prepared from *Hericium erinaceus* residue via mineral/protein purification and subsequent TEMPO-mediated oxidation. The structure, dispersity, and gelation ability of the prepared fungal nano chitin were studied. The results showed that the average length and width of the prepared fungal nano chitin were 336.6 nm and 6.4 nm, respectively, and the aspect ratio exceeded 50:1. The nano chitin retained the basic structure of chitin, while the crystallization index was improved. In addition, the dispersity of the nano chitin in aqueous media was evaluated by the effective diameter, and the polydispersion index was mainly affected by pH and ionic strength. Under acetic acid “gas phase coagulation”, the prepared nano chitin dispersions with mass concentrations of 0.2, 0.4, 0.6, and 0.8% were converted into gels by enhanced hydrogen bond crosslinking between nano chitins.

## 1. Introduction

Natural polymers, such as cellulose and chitin, have been widely designed as hydrogels [1,2], films [3,4], catalysts [5,6], and adsorbents [7,8] due to their abundant reserves. Chitin is the second most abundant biopolymer on earth after cellulose, and it is found in the shells of arthropods, the skeletons of squid and insects, and the cell walls of some fungi [9]. As one of the most important chitin derivatives, the highly crystalline nano chitin possesses numerous outstanding characteristics, such as a high aspect ratio, high surface area, favorable dispersity, renewability, biodegradability, and nontoxicity. These characteristics promote the wide application of nano chitin in composites [10,11,12,13], the food industry [14,15,16], water treatment [17], and biomedical fields [18,19].

Nano chitin, including chitin nanowhisker or nanofiber, is generally prepared from α- and β-chitin derived from the crab/shrimp shell or squid pen [20,21,22,23,24]. Although there are abundant crustacean wastes that can be used for chitin extraction, the quality of the extracted chitin is greatly influenced by seasonal factors and regional differences [25]. In contrast, extracting chitin from mushrooms can not only avoid the above problems, but also the extraction conditions are quite simple, because impurities, such as minerals, in mushrooms are not firmly bound to chitin. Many studies have shown that mushrooms are a promising resource for fungal chitin production [26,27,28,29]. Therefore, the exploration of fungal chitin from mushrooms for preparing nano chitin is meaningful and imperative. As one of the typical mushrooms in China, *Hericium erinaceus* is generally applied for polysaccharide production [30]. After the extraction of polysaccharides, the remained *Hericium erinaceus* residue can still be used for chitin extraction [27]. Therefore, the *Hericium erinaceus* chitin may be further applied to prepare nano chitin.

Nano chitin can be prepared from the polymer molecules by using a “bottom-up” process, such as electrospinning [31]. However, it is quite difficult to prepare nano chitin using this molecular assembly process. Alternatively, nano chitin can be also prepared by the “up-bottom” process, which is a process for extracting nanofibers from naturally occurring polymers. High crystallinity nano chitin prepared by chemical or mechanical methods belongs to this category. Recently, chitin nanofibers with diameters ranging from 20 to 200 nm were prepared by high-field ultrasonic treatment [32]. In addition, high-pressure homogenization technology has also been applied to obtain chitin nanofibers with uniform diameters [33]. Compared with these mechanical methods, the TEMPO-mediated oxidation method has become a preferred method and has made considerable progress in the past few years [34,35,36]. This is because the completion of the TEMPO method does not require complicated equipment, and highly crystalline nano chitin can be easily obtained through a simple chemical modification process.

Nano chitin can maintain a stable dispersion state in water and exhibits a special gelation behavior. The gelation of nano chitin is different from chitin because it does not involve a dissolution process. Compared with dissolved chitin, the dispersion of nano chitin has unique advantages of a large surface area, high aspect ratio, and high crystallinity [37], which are beneficial for preparing tough and self-standing hydrogels. Furthermore, the complicated dissolution process of chitin can be avoided. Recently, a strong self-supporting nano chitin hydrogel was successfully fabricated using TEMPO-oxidized nano chitin [38]. Additionally, both TEMPO/NaBr/NaClO and TEMPO/NaClO/NaClO_2_ systems have been applied to prepare nano chitins, which showed desirable gelation behaviors [36]. However, the above-mentioned nano chitins used for gel preparation were derived from crab (*Portunus trituberculatus*); the gelation of fungal nano chitin is still unknown. In this work, fungal nano chitin was extracted from *Hericium erinaceus* residue. The structure, crystallinity, and thermal stability of the prepared nano chitin were studied. The dispersion stability of the prepared nano chitin in an aqueous suspension was also investigated. Moreover, the prepared nano chitin dispersion was transformed into a gel under acidic conditions, and the gelation mechanism was preliminarily described.

## 2. Materials and Methods

### 2.1. Materials and Reagents

*Hericium erinaceus* fruiting body was sourced from Pingnan County (Ningde city, Fujian Province, China). The *Hericium erinaceus* residue used in this study was collected from the waste obtained after the extraction of polysaccharides from *Hericium erinaceus*. Tetramylpiperidone oxide (TEMPO) was supplied by Aladdin Reagent Co. Ltd. (Shanghai, China). Hydrogen chloride (HCl, 36%, *v*/*v*), sodium hydroxide (NaOH), sodium chloride (NaCl), sodium bromide (NaBr), sodium hypochlorite (NaClO), sodium chlorite (NaClO_2_), and acetic acid were purchased from Guangzhou Chemical Reagent Co., Ltd. (Guangzhou, Guangdong Province, China).

### 2.2. Preparation of Nano Chitin

The preparation of nano chitin from *Hericium erinaceus* residue was carried out using mineral/protein purification and subsequent TEMPO-mediated oxidation. Firstly, the raw materials were dispersed into an aqueous solution of 4 wt% hydrogen chloride at room temperature for 12 h to remove minerals. Secondly, the obtained residue was washed to neutral and then dispersed into an aqueous solution of 2 wt% sodium hydroxide at 85 °C for 3 h to remove proteins. After this step, the collected residue was washed to neutral and dispersed in an aqueous solution of 7.5 wt% sodium chlorite at 75 °C for 2 h. Finally, after the collected residue was washed to neutral, the fungal chitin was available.

To prepare the nano chitin, 0.25 g powdered fungal chitin was firstly suspended in 25 mL water containing 0.008 g TEMPO and 0.08 g sodium bromide. The oxidation reaction was started by adding 7.5 mL sodium hypochlorite solution (available chlorine > 7.5%). The pH of the reaction solution was maintained at 10 at room temperature by the addition of 2 wt% sodium hydroxide solution. The whole reaction was ended when the pH of the reaction solutions did not change any more. Subsequently, the suspensions were centrifuged at 7000 r/min for 10 min to collect the precipitate, which was then resuspended with the desired amount of distilled water for the next centrifugation. After the above centrifugal operation was repeated 3 times, appropriate amount of distilled water was added to the chitin to resuspend the precipitate and ultrasonically treated for 10 min. After sonication, the suspensions were centrifuged at 5000 r/min for 10 min to collect the upper liquid.

### 2.3. Preparation of Nano Chitin Gels

The nano chitin gels were prepared by acetic acid “gas phase coagulation”. Firstly, nano chitin dispersions with different concentrations (0.2 wt%, 0.4 wt%, 0.6 wt%, and 0.8 wt%) were poured into a small petri dish. Subsequently, the petri dishes with the nano chitin dispersions were placed in a larger petri dish, and then an appropriate amount of acetic acid was added to the larger petri dish such that it just covered the bottom of the petri dish. Finally, the larger petri dish was sealed overnight at room temperature to form the nano chitin gels.

### 2.4. Characterization Methods

The morphology of the prepared nano chitin was observed using an atomic force microscope (AFM; Santa Barbara, Veeco, Plainview, NY, USA) and a transmission electron microscope (TEM; JEM-1200EXII, JEOL, Tokyo, Japan). For AFM analysis, the nano chitin dispersion was dropped onto a fresh mica flake, dried in the air, and then used for AFM observation. For TEM analysis, the nano chitin dispersion was dropped onto electron microscope grids coated with a carbon-reinforced formvar film, which were then used for TEM observation. The basic structure of the nano chitin was characterized by Fourier transform infrared spectroscopy (FTIR) (Vector 33, Bruker, Germany) and X-ray diffraction (XRD) (D8 ADVANCE, Bruker, Germany). Before analysis, the freeze-dried samples were pressed into thin slices, which were used for FTIR and XRD analysis. The FTIR spectra were recorded from 500 to 4000 cm^−1^, while the XRD patterns were recorded at diffraction angles from 5° to 50°. The thermal stability of the nano chitin was analyzed by a thermal gravimetric analyzer (TGA; STA449 F3, Netzsch, Germany). The freeze-dried samples were examined by a thermo gravimetric analyzer under nitrogen gas from 35° to 500 °C with a heating rate of 10 °C/min. The particle size distribution, polydispersion index, and zeta potential of the nano chitin dispersions were measured by particle size and a zeta potentiometer (Omni, Brookhaven, GA, USA). The apparent viscosity of nano chitin was measured using a multifunctional rotary rheometer (ARES-G2, TA instrument, New Castle, DE, USA). The appearance of the nano chitin gels was photographed by a digital camera. The internal microscopic morphology of the nano chitin gels was photographed by a scanning electron microscope (SEM; EM-30AX, COXEM, Daejeon, Korea).

## 3. Results and Discussion

### 3.1. Characterization of Nano Chitin

**Figure 1:**
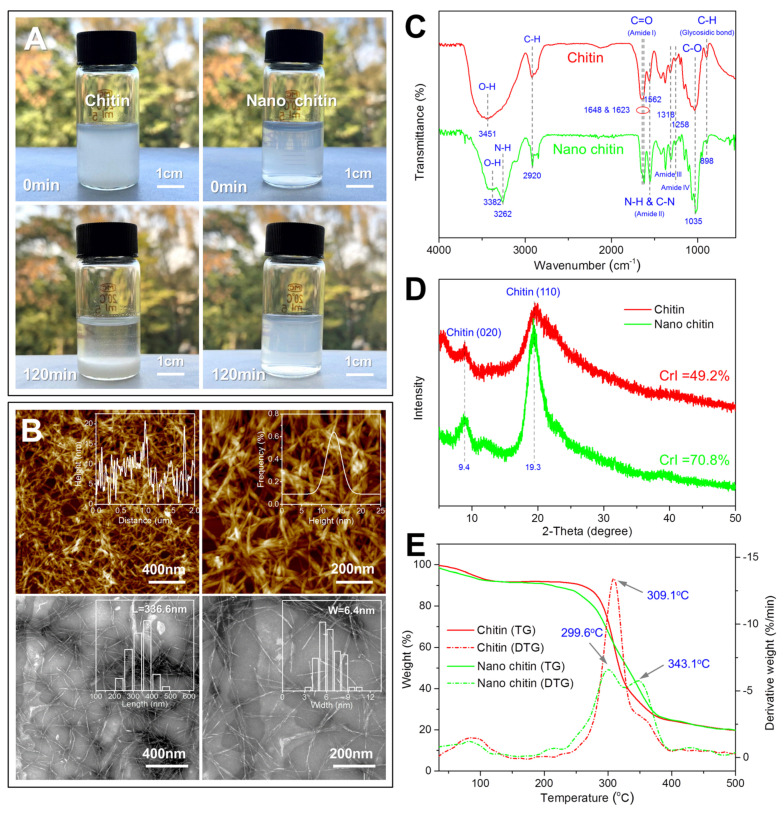
Optical photographs of chitin and nano chitin dispersed in water for 0 min and 120 min (**A**). AFM and TEM images of nano chitin (**B**). FTIR spectra (**C**), X−ray diffraction patterns (**D**), and TG−DTG curves (**E**) of chitin and nano chitin.

It is well known that chitin cannot be dissolved or dispersed in aqueous solution, while nano chitin can maintain a stable dispersion state in aqueous solution due to its strong electrostatic repulsion. In this work, the stability of the prepared chitin and nano chitin dispersed in water was investigated, and the results are shown in Figure 1A. As expected, the prepared chitin was not soluble in water and formed a suspension. After 120 min, all the chitin settled on the bottom of the bottle, indicating the poor dispersity of the chitin. In comparison, the prepared nano chitin formed a translucent dispersion in water, and there was no precipitation after standing for 120 min, indicating that the prepared nano chitin possessed good dispersity. In addition, the zeta potential of the prepared nano chitin dispersion was −27.8 mV, which was sufficient to maintain the stable dispersion state of the nano chitin. The obvious negative charge of the prepared nano chitin was mainly due to the selective formation of C-6 carboxylate groups on the chitin crystallite surfaces, which suggested that the prepared chitin was successfully oxidized in this work.

The microscopic morphology of the prepared nano chitin was observed by AFM and TEM, and the results are shown in Figure 1B. It can be seen from the AFM images that a large number of nano-scale fibrous chitin crystallites were entangled with each other and formed a network structure. The height variation and distribution of the nano chitin on the equator line of the AFM image were analyzed using the NanoScope Analysis software. It was found that the height of the prepared nano chitin varied greatly and was mainly distributed in the range of 5–20 nm. Therefore, there may have been a partial overlap of nano chitin in the AFM image. In order to obtain the aspect ratio of the prepared nano chitin, TEM was further used to observe the morphology of the nano chitin, and the obtained TEM images were analyzed using the Nano Measurer software. In accordance with the AFM images, fibrous nano chitin was observed. In addition, the length of the nano chitin was between 200 and 500 nm, while the width was between 3 and 11 nm. The average length and width were 336.6 nm and 6.4 nm, respectively, and the aspect ratio exceeded 50:1. Therefore, nano chitin with a high aspect ratio was successfully prepared from the *Hericium erinaceus* residue.

In order to determine the changes in the chemical structure of chitin after nanocrystallization, the FTIR characterization of chitin and nano chitin was carried out. As shown in Figure 1C, chitin showed a broad absorption peak at 3451 cm^−1^, which was attributed to the O-H stretching vibration [39]. In comparison, nano chitin showed two absorption peaks at 3382 cm^−1^ and 3262 cm^−1^, corresponding to O-H and N-H stretching vibrations, respectively. Both chitin and nano chitin showed a relatively weak absorption peak at 2920 cm^−1^, which was ascribed to C-H stretching vibration [40]. Chitin showed a single absorption peak at 1648 cm^−1^, which corresponds to the C=O stretching of the amide I group [41]. In addition, the absorption peaks at 1562 cm^−1^, 1318 cm^−1^, and 1258 cm^−1^ were found in both chitin and nano chitin, corresponding to amide II, amide III, and amide IV groups, respectively [42]. The absorption peaks at 1035 cm^−1^ and 898 cm^−1^ were also observed in both chitin and nano chitin, and were attributed to C-O stretching and C-H bond deformation, respectively [43,44]. In general, the chemical structure of the nano chitin was consistent with that of chitin, which suggested that TEMPO-mediated oxidation is a directional oxidation method.

In order to determine the changes in the crystal structure of chitin after nanocrystallization, chitin and nano chitin were characterized using X-ray diffraction. As shown in Figure 1D, both chitin and nano chitin showed obvious diffraction peaks at 2θ = 9.4° and 19.3°, corresponding to (020) and (110) crystal planes of chitin, respectively [45]. This result suggested that chitin retained its original crystal structure after TEMPO oxidation. Compared with chitin, it was found that these two characteristic diffraction peaks of nano chitin were obviously stronger. According to this result, the crystallization index of chitin and nano chitin was calculated based on the following formula [46]:

(1) $$\mathrm{CrI}\;(\%)=\frac{I_{110\;}{-\;I}_{\mathrm{am}}}{I_{110}}\;\times \;100\%\;$$

where *I*_110_ is the intensity of diffraction peak at 2θ = 20° and *I*_am_ is the intensity of amorphous diffraction at 2θ = 16°. The CrI of chitin and nano chitin was calculated as 49.2% and 70.8%, respectively. The increase in the crystallization index of chitin after nanocrystallization has also been also reported elsewhere [36]. This suggests that the nano-scale chitin forms a more orderly and compact crystal structure, which helps increase the hardness and strength of the chitin.

In order to determine the changes in the thermal stability of chitin after nanocrystallization, thermogravimetric analysis was performed on the chitin and nano chitin, and the results are shown in Figure 1E. It was observed from the TG curves that the weight loss of chitin and nano chitin could be divided into two stages. The first weight loss occurred between 35 and 100 °C, and was caused by the evaporation of residual moisture. The second stage of weight loss occurred between 250 and 400 °C, and was attributed to the degradation of chitin molecules. When the samples were heated to 500 °C, the chitin and nano chitin only retained 19.7% and 19.8% of their original weight, respectively. In addition, the chitin and nano chitin showed a similar residual weight after being heated to the high temperature, which indicated that the two samples were free of impurities and were basically composed of chitin molecules. On the other hand, it was also found from the DTG curves that the maximum degradation peak of chitin appeared at 309.1 °C, and the corresponding weight loss rate was 13.5%/min. However, nano chitin showed a degradation peak at 299.6 °C and 343.1 °C, and the corresponding weight loss rates were 6.9%/min and 5.9%/min, respectively. Therefore, both chitin and nano chitin exhibited desirable thermal stability.

### 3.2. Dispersion Stability of Nano Chitin

The dispersion stability of nano chitin plays a very important role in its subsequent gelation application, because well-dispersed nano chitin helps to form a uniform gel network structure. In general, the effective particle size of the nano chitin dispersions can directly reflect the formation of aggregates, while the polydispersity index (PDI) can reflect the uniformity of nano chitin distribution (the smaller the PDI, the more uniform the particle size distribution). In this work, the effects of different conditions, such as concentration, temperature, pH, and ionic strength, on the effective particle size and PDI of the prepared nano chitin dispersions were investigated.

**Figure 2:**
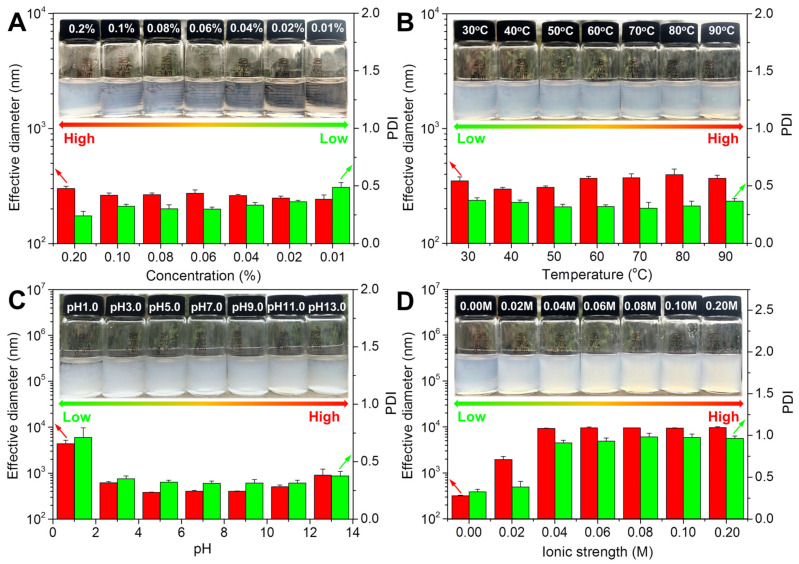
Effective diameter and PDI of nano chitin prepared from *Hericium erinaceus* residue at different concentrations (**A**), temperatures (**B**), pH values (**C**), and ionic strengths (**D**).

The effect of concentration on the nano chitin dispersions is shown in Figure 2A. The concentration had an obvious effect on the appearance of the prepared nano chitin dispersions. As the concentration decreased from 0.2% to 0.01%, the nano chitin dispersions gradually changed from translucent to transparent. In addition, the effective particle size of all the nano chitin dispersions was maintained at about 300 nm, indicating that no chitin aggregates were formed during the concentration change. On the other hand, the concentration showed little influence on the PDI of the nano chitin dispersions, except for the 0.01% concentration, for which the corresponding PDI was close to 0.5. This result suggested that the nano chitin dispersions may have been unevenly distributed in the water at extremely low concentrations. The reason for this might be that as the amount of nano chitin per unit volume decreases, the complete dispersion of nano chitin per unit volume of water becomes more difficult.

The effect of temperature on the nano chitin dispersions is depicted in Figure 2B. There were no obvious differences in the dispersion state of the nano chitin in the temperature range of 30–90 °C, and the corresponding effective particle size and PDI were maintained at around 300 nm and 0.35, respectively. Therefore, under these temperatures, the chitin suspensions showed neither the visible aggregation nor the uneven distribution of nano chitin.

The effect of pH on the prepared nano chitin dispersions is shown in Figure 2C. It was found that pH showed an obvious influence on the appearance of the prepared nano chitin dispersions. The nano chitin dispersions exhibited good dispersion properties at pH levels of 3.0, 5.0, 7.0, 9.0, and 11.0, and the corresponding effective diameter and PDI were maintained within 600 nm and 0.35, respectively. When the pH decreased to 1.0 or increased to 13.0, obvious floccules appeared in the nano chitin dispersions, with effective diameters of 4400 nm and 900 nm, and PDIs of 0.8 and 0.4, respectively. These results suggested that acidic conditions had a greater effect on the stability of the nano chitin. The decreased colloid stability of nano chitin under acidic conditions is likely attributed to the protonation of the –COO^−^ group on the nano chitin surface [47].

The effect of ionic strength on the prepared nano chitin dispersions is shown in Figure 2D. It can be observed from the illustration that the ionic strength showed an obvious influence on the appearance of the nano chitin dispersions. In the absence of NaCl, the nano chitin dispersions were uniform and translucent. As the ionic strength increased, the turbidity of the nano chitin dispersions gradually increased. In addition, the effective diameter and PDI of the prepared nano chitin dispersions were also affected by the ionic strength. Without the addition of NaCl, the effective diameter and PDI of the nano chitin dispersions were 300 nm and 0.35, respectively, indicating favorable dispersion and colloidal stability. When the applied ionic strength was only 0.02 M, the effective diameter and PDI of the nano chitin dispersion reached 1900 nm and 0.38, respectively, indicating the formation of aggregates. When the ionic strength was increased to 0.04 M, 0.06 M, 0.08 M, 0.1 M, and 0.2 M, the effective diameter and PDI of the corresponding nano chitin dispersions further increased. This indicated that the increase in ionic strength caused the nano chitin dispersion to aggregate in water. Due to the presence of additional Na^+^ and Cl^−^, the van der Waals gravitational force will exceed the electrostatic repulsion and dominate the interaction between nano chitin, leading to the formation of flocculation in the nano chitin dispersions [47].

### 3.3. Gelation of Nano Chitin

**Figure 3:**
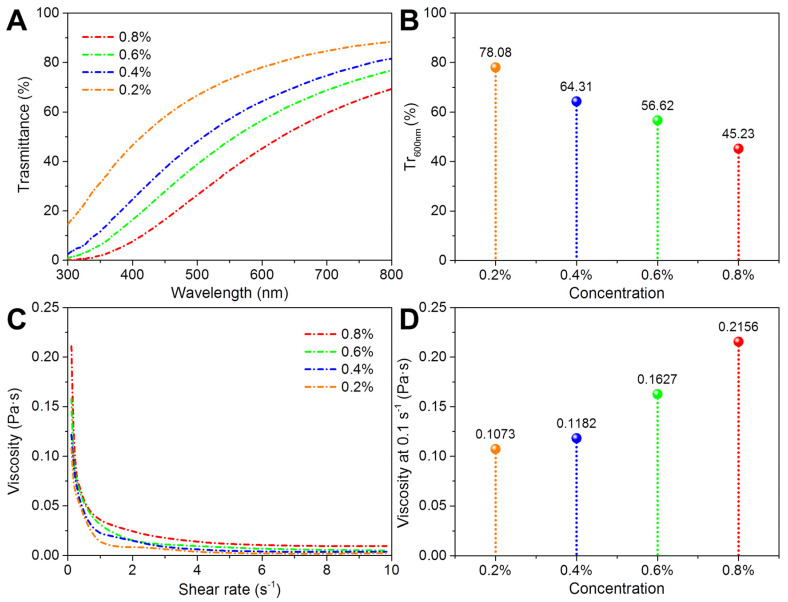
Light transmittance scanning curves (**A**), light transmittance at 600 nm (**B**), apparent viscosity shear curves (**C**), and the apparent viscosity at 0.1 s^−1^ (**D**) of the prepared nano chitin dispersions with different concentrations.

In this work, the gelation ability of the prepared nano chitin was analyzed using nano chitin dispersions with mass concentrations of 0.2%, 0.4%, 0.6%, and 0.8%. Before gelation, the light transmittance and apparent viscosity of the nano chitin dispersions were analyzed. As shown in Figure 3A, the light transmittance of all the prepared nano chitin dispersions increased with the scanning wavelength. Furthermore, it can be found from Figure 3B that the light transmittance of the nano chitin dispersions at 600 nm decreased from 78.08% to 45.23% as the concentration increased from 0.2% to 0.8%. It can be inferred that the higher the concentration of the nano chitin dispersion, the lower the light transmittance of the subsequently formed nano chitin gel. The shear viscosity scan curves of the prepared nano chitin dispersions are displayed in Figure 3C. The apparent viscosity of all samples exhibited typical shear thinning behavior. When the shear rate increased from 0.1 to 1.0 s^−1^, the apparent viscosity of the nano chitin dispersions dropped sharply, showing pseudoplastic fluid behavior. Subsequently, the apparent viscosity of the nano chitin dispersions stabilized when the shear rate was increased from 1.0 to 10 s^−1^. The apparent viscosity of the prepared nano chitin dispersions at 0.1 s^−1^ is also compared and depicted in Figure 3D. The nano chitin dispersions with higher concentrations showed higher apparent viscosity. The larger apparent viscosity helped to increase the interaction and crosslinking probability between nano chitins, which is more favorable for the formation of gels.

**Figure 4:**
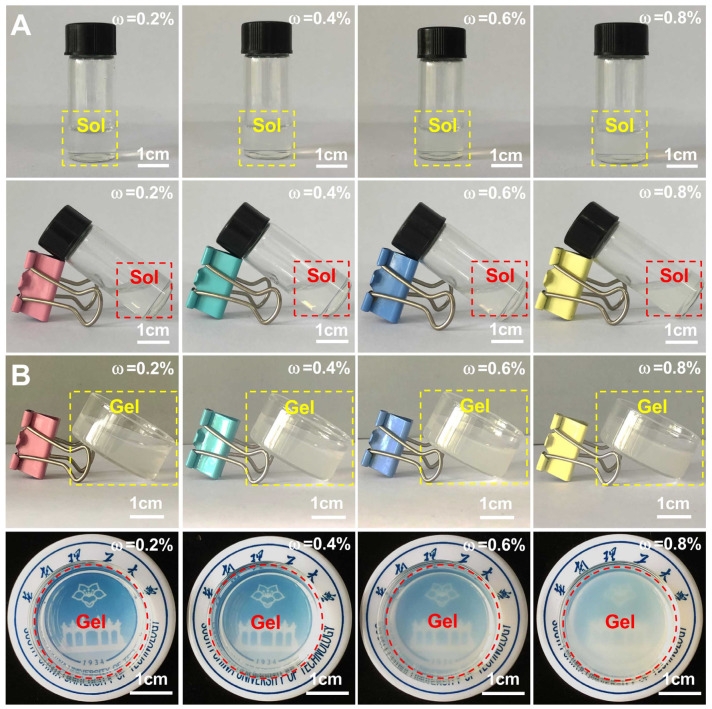
Optical photographs of the prepared nano chitin dispersions at different concentrations (**A**) and the corresponding nano chitin gels (**B**).

Based on the gas phase coagulation method, the nano chitin dispersions were used to prepare nano chitin gels. As depicted in Figure 4A, the liquid level in the serum bottle changed after tilting the vertical nano chitin dispersions, indicating that the nano chitin dispersions were in a fluid state before gas phase coagulation. As shown in Figure 4B, all samples did not exhibit obvious liquid level changes after being placed obliquely after the gas phase coagulation, indicating that the nano chitin dispersions with concentrations of 0.2%, 0.4%, 0.6%, and 0.8% formed nano chitin gels. Previous researchers have prepared nano chitin gels using nano chitin dispersions with mass concentrations of 0.4% and 1.0% [36,38]. In this work, nano chitin gels with a broad concentration range were successfully prepared, and the prepared nano chitin was derived from a new type of fungal resource—*Hericium erinaceus*. It is worth noting that there was no obvious shrinkage and deformation of the prepared nano chitin gels, indicating the desirable gelation ability of the nano chitin dispersions. However, the prepared nano chitin gels were fragile in a swollen state, which may be related to the high swelling degree of the gels. In addition, it was found that as the mass concentration of the nano chitin dispersions increased from 0.2% to 0.8%, the transparency of the corresponding nano chitin gels gradually decreased. This suggested that the gel network became denser as the concentration of nano chitin increased. Based on the above results, nano chitin prepared from *Hericium erinaceus* residue can be regenerated from the nano dispersed state into a new natural polymer hydrogel by the acid-induced gas phase coagulation method.

**Figure 5:**
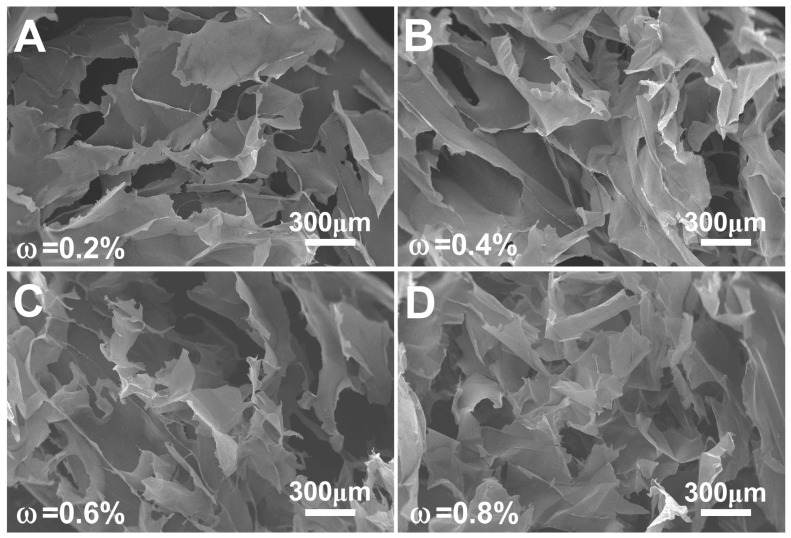
Internal structure of the prepared nano chitin gels with concentrations of 0.2% (**A**), 0.4% (**B**), 0.6% (**C**), and 0.8% (**D**).

The internal morphology of the prepared nano chitin gels was observed by SEM, and the results are shown in Figure 5. It was observed that the internal morphology of the nano chitin gels did not show an ordered three-dimensional porous network structure, but rather showed an irregular internal morphology. This internal morphology was mainly composed of the gel walls that were formed by the aggregation of nano chitin. The gel walls also exhibited a very loose texture, accompanied by macropores with sizes of tens to hundreds of microns. These pores were formed by the loss of moisture inside the gels after being freeze-dried.

**Figure 6:**
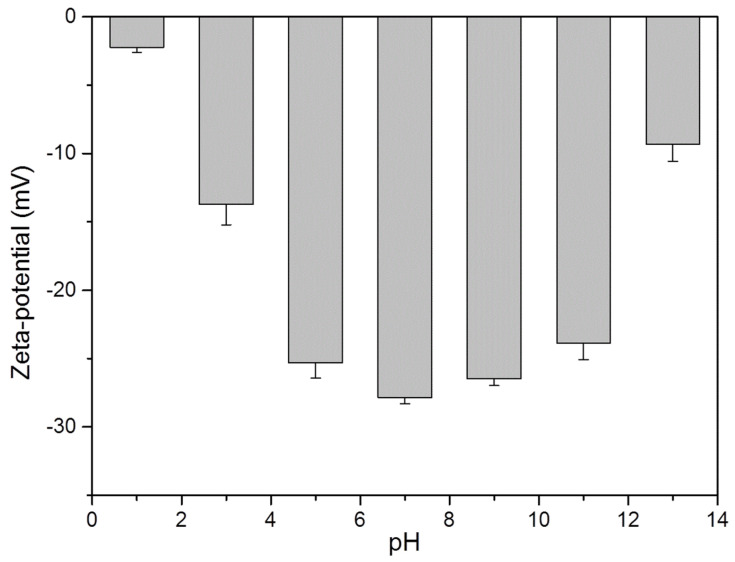
Zeta potential of nano chitin dispersions at different pH.

In order to explore the essential reason for why nano chitin can form a gel under acidic conditions, the zeta-potential of nano chitin at different pH values was measured. As shown in Figure 6, the nano chitin had the highest zeta potential at pH 7.0, indicating that it had the best dispersibility under neutral conditions. However, the corresponding zeta potential decreased regardless of whether the pH was increased or decreased, indicating that both alkaline and acidic environments affected the charge of the nano chitin dispersions. When the pH dropped to 1.0, the corresponding zeta-potential was only −2.2 mV, the smallest of the tested pH values. Therefore, acidic conditions had the greatest effect on the dispersibility of the prepared nano chitin. Under acidic conditions, the –COO^−^ group on the molecular chain of nano chitin will be converted into –COOH, thereby reducing the negative charge of nano chitin. The weakening of electrostatic repulsion leads to the aggregation of nano chitin, which enhances the interaction between chitin molecules, thereby achieving the gelation of nano chitin through enhanced intermolecular hydrogen bond crosslinking.

## 4. Conclusions

In conclusion, nano chitin with a high aspect ratio was successfully prepared from *Hericium erinaceus* residue. The prepared nano chitin retained the basic structure of chitin; however, its crystallization index was obviously improved. Moreover, the prepared nano chitin could be well-dispersed in aqueous solution, and the dispersity was affected by pH and ionic strength. More importantly, the prepared nano chitin dispersion had a desirable gelation ability and could be converted into gels by an acid-induced gas phase coagulation method. Therefore, similar to other natural polysaccharide gels, the prepared nano chitin gels may serve as a promising candidate for a wide range of applications, such as tissue engineering, drug delivery, and pollutant removal.

## Data Availability

The data presented in this work are available on request from the corresponding author.
